# Discriminating geographical origins of green tea based on amino acid, polyphenol, and caffeine content through high‐performance liquid chromatography: Taking Lu’an guapian tea as an example

**DOI:** 10.1002/fsn3.1062

**Published:** 2019-05-16

**Authors:** Huan Su, Weiquan Wu, Xiaochun Wan, Jingming Ning

**Affiliations:** ^1^ State Key Laboratory of Tea Plant Biology and Utilization Anhui Agricultural University Hefei China; ^2^ Anhui Lu’an Guapian Tea Industry Co., Ltd Lu’an China

**Keywords:** factor selection, geographical origin discrimination, high‐performance liquid chromatography, Lu’an guapian tea, pattern recognition

## Abstract

Seventy‐three Lu'an guapian tea (LAGP) samples were collected from 11 growing locations in the city of Lu'an, Anhui Province, China. Through high‐performance liquid chromatography, 18 amino acids, along with gallic acid, caffeine, and five catechins, were quantitatively detected. Hierarchical cluster, correlation and principal component analysis, and a support vector machine were used for geographical discrimination. The findings suggested that the differences in tea quality between the inner and outer mountain regions are related to isoleucine, leucine, phenylalanine, and valine contents, with a correlation coefficient of more than 0.85. Principal component analysis combining with support vector machine was a feasible method. The identification rates for the inner and outer mountains were 97.96% in the training set and 95.83% in the prediction set. Furthermore, the identification rates for the three counties were 91.84% and 95.83% in the training and prediction sets, respectively.

## 1 INTRODUCTION

Green tea, the most widely consumed tea in China, can benefit human health because of its chemical composition, which includes catechins, theanine, and caffeine (Cooper, 2012; Dulloo, Seydoux, Girardier, Chantre, & Vandermander, 2000; Sinija & Mishra, 2008). The chemical composition of tea depends on numerous factors, such as planting area, tea plantation management, processing practices, tea variety, and climate. However, the planting area plays the most important, direct role in the characterization and differentiation of tea quality and hence their market prices. Therefore, to evaluate tea quality, effective identification of geographical origin is crucial.

Lu'an guapian tea (LAGP), produced in Lu'an, Anhui Province, and the top 10 most famous teas in China (Bai et al., 2017), is the only tea in which tender leaves are processed without any buds or stems (Heiss, 2001). Due to the special process involved in its production, LAGP has a chestnut or even orchid‐like aroma as well as a taste stronger than normal green tea. The area used for its cultivation has greatly expanded in the past few years.

Lu'an guapian tea development has, however, resulted in several problems, mostly regarding the lack of standards and increase in market irregularities (Zhan, Liu, & Yu, 2017). Differences in the prices of LAGP from different origins can be extreme, which could lead to the market confusion. In 2008, LAGP was awarded geographical indication by the General Administration of Quality Supervision, Inspection, and Quarantine of the People's Republic of China with the region of origin being the border areas of Jinzhai County, Yu'an District, and Huoshan County (administrative levels of district and county are equal, but with different features in city establishment in China), covering the entire city of Lu'an. However, the core region of LAGP only existed in the inner mountain not the outer. The inner mountain region covers Qishan Village, Xiang Hongdian Reservoir, Flowers Hill, and Gongdian Village in Jinzhai County; Dushan Town, Shuangfeng, Shi podian Town, and Shajia Cove in Yu'an District; and Zhufo Monastery Town in Huoshan County. The main outer mountain region includes Shi Banchong in Yu'an District's Lion Village (Fan, Xiaopei, Wang, & Han, 2017). At present, most technique for tea identification (quality and variety) relies on sensory evaluation of color, aroma, taste, and appearance by the trained tea specialists. The obvious disadvantage about the sensory evaluation could be easily influenced by the taster's experience or environment. Teas from the same geographical origin and with the same processing method probably have a similar or typical composition. And such kind of chemical composition could afford their distinctive characteristics and also enable LAGP to be discriminated according to the geographical origins.

To determine the differences in chemical compositions, chemical analysis (such as spectroscopy and chromatography) has been widely applied to tracing the tea origins. Chemical components have been identified using proton nuclear magnetic resonance and near‐infrared spectroscopy (Li, Jin, Sun, Ye, & Liu, 2019; Meng et al., 2017), gas chromatography–mass spectrometry (Luo, Chen, Gao, Liu, & Wu, 2017), electronic tongue (Huo et al., 2014), and electronic nose (Chen, Liu, Zhao, & Ouyang, 2013). High‐performance liquid chromatography (HPLC) could classify six tea categories (Ning et al., 2017) and green tea from China and Korea (Jang et al., 2008). Combined with discriminate analysis, HPLC has also been applied for authentic origins discrimination of many agricultural products, such as ginger (Yudthavorasit, Wongravee, & Leepipatpiboon, 2014), wine (Rastija & Srečnik, 2009), honey (Hermosín, Chicón, & Dolores Cabezudo, 2003), and olive oil (Cunha, Amaral, Fernandes, & Oliveira, 2006). Compared with secondary metabolites (e.g., catechins), the chemical properties of amino acids are more stable in green tea (Ananingsih, Sharma, & Zhou, 2013). Some relationship between the quality of green tea and amino acid content has been demonstrated (Ding, Yu, & Mou, 2002).

Total free amino acid, catechin, and caffeine contents account for approximately 20% of the dry weight of green tea. More accurate and available information can be provided by increasing the number of analyzed samples and combining this information with other analytical data and statistical treatment of the results (Hermosín et al., 2003). This study aimed to fill the knowledge gap regarding the chemical composition of LAGP. Besides, the results were examined to establish whether the chemical components of tea can discriminate its geographical origin. The following amino acids and catechins are referred to in this paper: alanine (Ala), arginine (Arg), aspartic acid (Asp), cysteine (Cys), glutamic acid (Glu), glycine (Gly), histidine (His), isoleucine (Ile), leucine (Leu), methionine (Met), lysine (Lys), phenylalanine (Phe), proline (Pro), serine (Ser), threonine (Thr), tyrosine (Tyr), valine (Val), theanine (Thea), gallic acid (GA), (−)‐epigallocatechin (EGC), (−)‐epigallocatechin gallate (EGCG), (+)‐catechin (C), (−)‐epicatechin (EC), and (−)‐epicatechin gallate (ECG).

## 2 MATERIALS AND METHODS

### 2.1 Samples and chemicals

**Figure 1:**
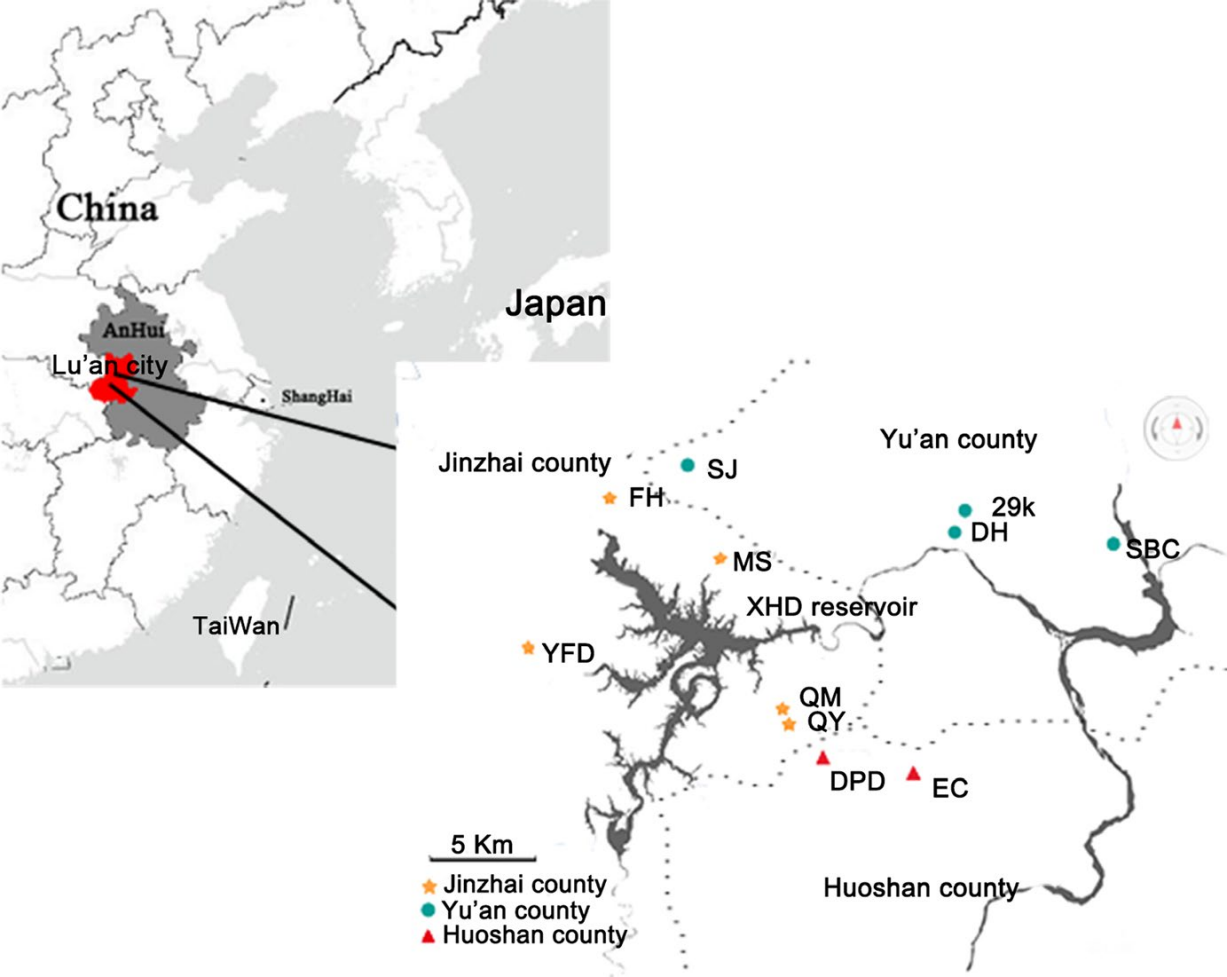
Sample collection areas

In total, 73 LAGP samples were collected from 11 village points, two counties, one district, and the inner and outer mountain regions (Figure 1); the latitude and longitude of the map center is 116.12°N, 31.51°E (Google Maps). All tea samples were processed by tea farmer using the first to third leaf of the population species according to the techniques outlined in the Anhui provincial standard for LAGP DB34/T237–2017, and the author took samples once per day from 18 April 2017 to 24 April 2017. The name, altitude, and sample size of the sampling point are shown in Table S1. The main difference between inner mountain tea and outer mountain tea is the altitude of the plantation. The samples were kept in aluminum foil bags, stored in a freezer at 4°C, and ground into powder before being strained using a 600‐μm sieve for subsequent analysis.

High‐performance liquid chromatography‐grade acetonitrile was obtained from Tedia (Tedia Co.); GA, EGC, EGCG, C, EC, ECG (˃99.99%), caffeine, and Thea were purchased from Sigma. A reagent kit was obtained from Waters that included a mixture of 17 amino acid hydrolysate standards (Waters AccQ·Tag Amino Acid Standard H), the derivatization reagent AQC (Waters AccQ·Fluor Reagent), and eluent A (Waters AccQ·Tag Eluent A, concentrate). Milli‐Q‐treated water was prepared to purity higher than 18 MΩ cm using a water purification system (Aquapro International Co.).

### 2.2 Test solution preparation

#### 2.2.1 Amino acids

According to the ISO/WD 19563 (2017), 1.00 ± 0.01 g of sample powder in a 200‐ml beaker and 100 ml of boiling water were added. The sample was brewed on a magnetic stirrer (500 upm) for 5 min, filtered, and made up to a certain volume. Approximately, 1 ml of the sample solution was centrifuged at 16,000 g(rcf) for 10 min. And 10 μl of the extract was reacted with 90 μl derivatization reagent under 55°C to perform the precolumn derivatization (Reverter, Lundh, & Lindberg, 1997).

#### 2.2.2 Polyphenols and caffeine

According to the ISO 14502‐2: 2005 (2006), 0.20 ± 0.01 g of powder was eluted in magnetic stirring apparatus (70°C) with 5 ml of methanol–water (70%) for 10 min. Then, it was centrifuged at 4,200 g(rcf) for 10 min and transferred into a 10‐ml volumetric flask. Repeat extraction again and mix the two extracts to 10 ml. The extract was filtered through a 0.22‐μm Millipore membrane before injection.

### 2.3 Chromatography conditions

**Figure 2:**
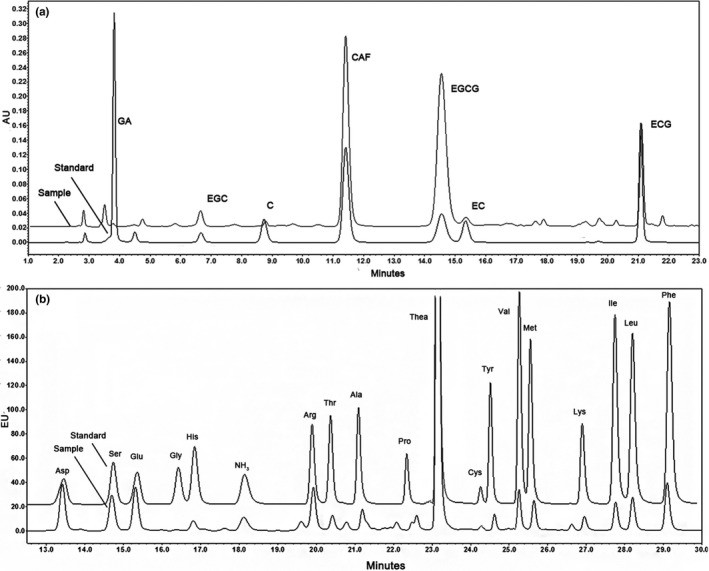
High‐performance liquid chromatography (HPLC) chromatogram of catechins, caffeine, and amino acids. HPLC chromatograms of the mixed references of amino acids (a) and of catechins and caffeine (b)

Quantitative analysis of amino acids was performed on a Waters e2695 series HPLC system equipped with a Waters 2475 fluorescence detector (*λ*ex = 250 nm, *λ*em = 395 nm) and Waters AccQ·Tag reversed‐phase HPLC column (4 µm, 3.9 × 150 mm, 37°C); Empower (version 3.0) was used for reporting and processing chromatographic information. The mobile phase comprised 1:11 (v/v) AccQ·Tag Eluent A and 10:11 (v/v) Milli‐Q water (A), acetonitrile (B), and Milli‐Q water (C). The gradient conditions of the mobile phase were as follows (1.0 ml/min): 17 min, 100% A turned to 91% A, 5% B; 24 min, 80% A, 17% B; 32–34 min, 68% A, 20% B; 35 min, 0% A, 40% B; 37 min, 0% A, 60% B; 38 min, returned to 100% A and maintained for 7 min to clear and stabilize the column. The peak retention times were displayed in the software (Figure 2a).

Polyphenols and caffeine were detected on a Waters 2489 Ultraviolet‐Visible Detector (*λ* = 278 nm) and the analytical column (Phenomenex C_18_, 5 μm, 250 × 4.6 mm, 35°C). The mobile phase consisted of solvents A (9% acetonitrile, 2% acetic acid, 0.2% EDTA, water) and B (80% acetonitrile, 2% acetic acid, 0.2% EDTA, water), followed by ISO 14502‐2: 2005 (2006) (ISO). The gradient conditions of the mobile phase were as follows (1.0 ml/min): 0–10 min, 100% A; 25–35 min, 68% A; 40–45 min, 45% A; 50–60 min, 100% A. The peak retention times and areas are shown in the software (Figure 2b).

### 2.4 Statistical analysis

In this study, hierarchical cluster, correlation and principal component analysis were used for factors selecting. Hierarchical cluster analysis (HCA), an unsupervised learning process, divides similar objects into groups or more subsets through static classification, and members in the same subset have similar properties, which use Euclidean distance (Saito & Toriwaki, 1994) to calculate the distance (similarity) between different categories of data points. A more general alternative is the weighted Euclidean distance between two vectors *x_i_* and *x_j_*,

(1) $$d_{i,j}={\left[\sum_{k=1}^{\text{K}}w_{k}{\left(x_{i,k}-x_{j,k}\right)}^{2}\right]}^{1/2}$$

For *w_k_* = 1 for each *k* = 1, … , K, Equation (1) reduces to the ordinary Euclidean distance.

Correlation analysis refers to the analysis of two or more related variable elements to measure the closeness of the two variable factors (Ezekiel, 1941). The Pearson correlation coefficient, the covariance of the two variables divided by the product of their standard deviations, was used to measure the degree of correlation in this study. Principal component analysis (PCA) (Wold, Esbensen, & Geladi, 1987; Yu, Wang, Xiao, & Liu, 2009), the simplest method for analyzing multivariate statistical distributions with feature variables, is often used to reduce the dimensionality of data sets while maintaining the maximum contribution of the variance within. The obtained results provide a rough classification of all samples.

Support vector machine (SVM) (Vapnik, 2013) is a kind of generalized linear classifier that classifies data according to supervised learning. And the decision boundary is the maximum margin hyperplane for solving learning samples. The SVM can be nonlinearly classified by nuclear method and is one of the common nuclear learning methods, which uses the hinge loss function to calculate the empirical risk and adds a regularization term to the solution system to optimize the structural risk (Chen, Zhao, Fang, & Wang, 2007). Moreover, SVM can learn in high‐dimensional feature space with fewer training data.

The all‐for‐mentioned statistical analyses were performed using Matlab 2016a (MathWorks, Inc.). Correlation analysis and one‐way ANOVA were performed using SPSS (version 22.0 for Windows).

## 3 RESULTS AND DISCUSSION

### 3.1 Amino acid, GA, catechin, and caffeine detection and analysis

**Figure 3:**
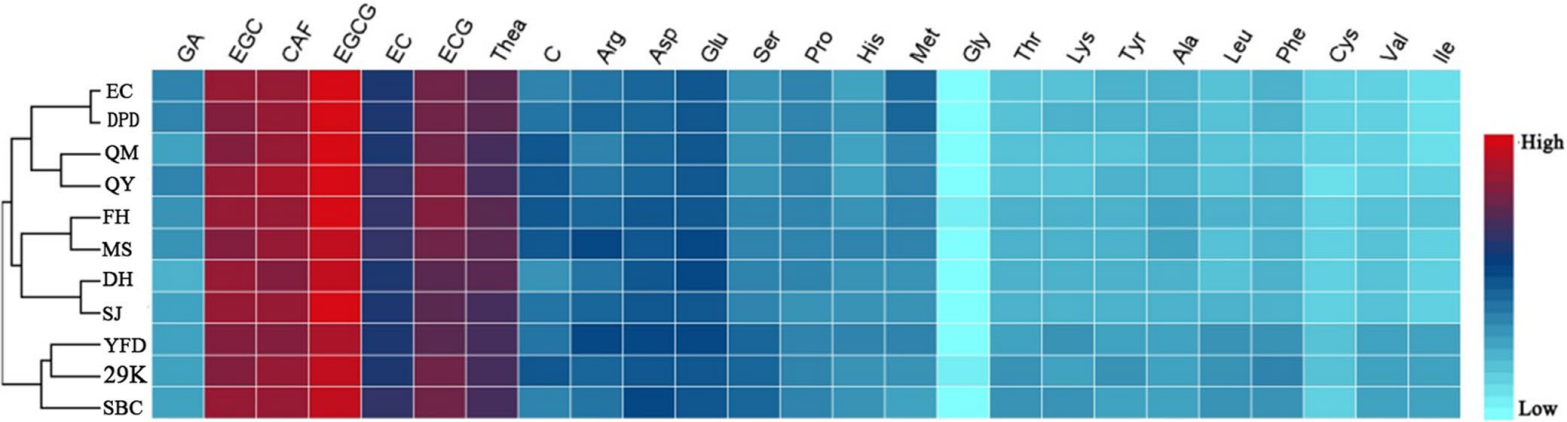
Heat map of 25 variables and the results of hierarchical clustering for origins

In this study, the contents of all tested substances were quantified using HPLC and provided in Table S2. This study examined two classes of tea: inner mountain LAGP (IMGP) and outer mountain LAGP (OMGP, including YFD, 29K, and SBC). Table 1 shows the differences in the three major metabolite contents for each area. It was remarkable that the concentrations of GA, Met, Leu, Ile, and Val had significant differences (*p* < 0.05) among HSGP, JZGP, and YAGP; and the difference was almost 16%–60% between IMGP and OMGP in contents of EGCG, Ser, Lys, Phe, Val, etc. A heat map (Figure 3) was created using HemI (version 1.0) to visualize the content composition in different regions: Red, cyan, and blue areas indicate high, low, and moderate levels of chemical composition, respectively. All LAGP had the same relative contents: high EGCG, caffeine, EGC, ECG, and Thea but low Gly and Cys contents. Hierarchical cluster analysis, an unsupervised learning process, divides similar objects into groups or more subsets through static classification, and members in the same subset have similar properties, which use Euclidean distance to calculate the distance (similarity) between different categories of data points. Huoshan County guapian (HSGP; EC and DPD) and OMGP exhibited great difference with the other regions as shown in HCA.

**Table 1:** The content of each substance at different regions (WT%, means ± *SD*)

	Group 1			Group 2	
	HSGP	JZGP	YAGP	IMGP	OMSP
GA	0.049 ± 0.002^c^	0.036 ± 0.002^b^	0.027 ± 0.002^a^	0.036 ± 0.002	0.033 ± 0.002
EGC	3.230 ± 0.173^b^	2.773 ± 0.124^a^	3.328 ± 0.131^b^	3.095 ± 0.105	2.945 ± 0.144
C	0.062 ± 0.008^a^	0.122 ± 0.008^b^	0.073 ± 0.010^a^	0.090 ± 0.008	0.105 ± 0.010
CAF	3.445 ± 0.117^ab^	3.785 ± 0.133^b^	3.196 ± 0.103^a^	3.492 ± 0.101	3.572 ± 0.131
EC	0.492 ± 0.024^a^	0.512 ± 0.022^a^	0.534 ± 0.025^a^	0.500 ± 0.014	0.556 ± 0.033
EGCG	11.069 ± 0.510^b^	8.718 ± 0.373^a^	8.023 ± 0.260^a^	9.275 ± 0.315	7.933 ± 0.280
ECG	1.805 ± 0.070^b^	1.939 ± 0.085^b^	1.413 ± 0.040^a^	1.750 ± 0.068	1.672 ± 0.061
Asp	0.109 ± 0.004^a^	0.147 ± 0.009^b^	0.156 ± 0.008^b^	0.137 ± 0.006	0.159 ± 0.010
Ser	0.036 ± 0.001^a^	0.059 ± 0.005^b^	0.071 ± 0.006^b^	0.051 ± 0.003	0.079 ± 0.007
Glu	0.149 ± 0.004^a^	0.170 ± 0.008^a^	0.165 ± 0.009^a^	0.163 ± 0.006	0.167 ± 0.010
Gly	0.003 ± 0.000^a^	0.003 ± 0.000^a^	0.003 ± 0.000^a^	0.003 ± 0.000	0.004 ± 0.000
His	0.031 ± 0.001^a^	0.039 ± 0.002^a^	0.039 ± 0.003^a^	0.036 ± 0.002	0.042 ± 0.003
Arg	0.094 ± 0.006^ab^	0.123 ± 0.012^b^	0.088 ± 0.006^a^	0.098 ± 0.007	0.123 ± 0.013
Thr	0.015 ± 0.000^a^	0.019 ± 0.001^a^	0.025 ± 0.002^b^	0.017 ± 0.001	0.028 ± 0.002
Ala	0.017 ± 0.001^a^	0.021 ± 0.001^b^	0.022 ± 0.001^b^	0.019 ± 0.001	0.024 ± 0.001
Pro	0.051 ± 0.002^a^	0.054 ± 0.002^a^	0.053 ± 0.002^a^	0.051 ± 0.002	0.057 ± 0.002
Cys	0.009 ± 0.002^a^	0.009 ± 0.002^a^	0.011 ± 0.002^b^	0.009 ± 0.002	0.011 ± 0.002
Tyr	0.017 ± 0.002^a^	0.020 ± 0.002^a^	0.026 ± 0.002^b^	0.019 ± 0.002	0.027 ± 0.002
Val	0.008 ± 0.002^a^	0.015 ± 0.002^b^	0.021 ± 0.002^c^	0.013 ± 0.002	0.023 ± 0.002
Met	0.097 ± 0.002^c^	0.053 ± 0.002^b^	0.037 ± 0.002^a^	0.061 ± 0.002	0.039 ± 0.002
Lys	0.016 ± 0.002^a^	0.020 ± 0.002^ab^	0.024 ± 0.002^b^	0.018 ± 0.002	0.027 ± 0.002
Ile	0.007 ± 0.002^a^	0.012 ± 0.002^b^	0.018 ± 0.002^c^	0.011 ± 0.002	0.020 ± 0.002
Leu	0.012 ± 0.002^a^	0.018 ± 0.00^b^	0.024 ± 0.002^c^	0.016 ± 0.002	0.026 ± 0.002
Phe	0.016 ± 0.002^a^	0.021 ± 0.002^a^	0.032 ± 0.002^b^	0.019 ± 0.002	0.035 ± 0.002
Thea	1.076 ± 0.002^a^	1.003 ± 0.002^a^	0.993 ± 0.002^a^	0.999 ± 0.002	1.045 ± 0.002

Note

Values in the same row of Group 1 that are labeled with different superscript letters (a–c) differ significantly (*p* < 0.05). Statistical analysis was one‐way ANOVA with pairwise post hoc comparisons by the method of Bonferroni.

### 3.2 Correlation analysis

Correlation analysis between chemical composition and counties/districts based on Pearson correlation coefficient was used, and the Pearson correlation coefficient is shown in Table 2. The value was between +1 and −1, where the numerical value indicates the correlation strength, and “+” or “−” indicates the positive or negative correlation. The variables strongly correlated with IMGP and OMGP were Phe, Leu, Ile, and Val, with all correlation coefficients being >0.85, whereas those strongly correlated with Jinzhai County LAGP (JZGP), Yu'an District LAGP (YAGP), and HSGP were GA and Met, with all correlation coefficients being >0.5. The HCA results afforded great similarity among the LAGP types. A study of the found components revealed that the main bitter amino acids in the tea included L‐Ile, L‐Leu, L‐Tyr, L‐Phe, and L‐Val; the more hydrophobic the R group of the amino acid side chain is, the more bitter the taste is (Mao et al., 2018). GA content is significantly positively correlated with the quality grade of green tea, and GA is a characteristic component of green tea (Graham, 1992). These findings match with the results of sensory evaluation: Inner mountain tea has a less bitter, mellower taste than outer mountain tea does. However, the geographical tracing was negatively correlated with the geographical distance. Some sampling points, such as SJ and FH as well as QY and FH, border each other, and sometimes, they are even two sides of the same mountain. For example, the place adjacent to Jinzhai County and Huoshan County is the famous Dabie Mountains.

**Table 2:** Correlation analysis for chemical composition and region

	GA	EGC	C	CAF	EC	EGCG	ECG	Asp	
Group 1	−0.319**	−0.250*	−0.139	−0.317**	−0.039	−0.527**	−0.451**	0.538**	
Group 2	−0.575**	0.130	−0.041	−0.208	0.126	−0.467**	−0.405**	0.313**	
	Ser	Glu	Gly	Phe	His	Arg	Thr	Ala	
Group 1	0.811**	0.125	0.221	0.877**	0.346**	0.191	0.830**	0.536**	
Group 2	0.405**	0.098	0.145	0.430**	0.163	−0.110	0.464**	0.363**	
	Pro	Cys	Tyr	Val	Met	Lys	Ile	Leu	Thea
Group 1	0.367**	0.519**	0.758**	0.887**	−0.471**	0.820**	0.897**	0.893**	−0.131
Group 2	0.042	0.270*	0.456**	0.481**	−0.779**	0.370**	0.484**	0.450**	−0.136

Note

Pearson correlation coefficient was used for analysis. Group 1 means IMGP and OMGP; Group 2 means JZGP, YAGP, and HSGP.

* Correlation is significant at a 0.05 level (two‐tailed);

** Correlation is significant at a 0.01 level (two‐tailed).

### 3.3 Principal component analysis

**Figure 4:**
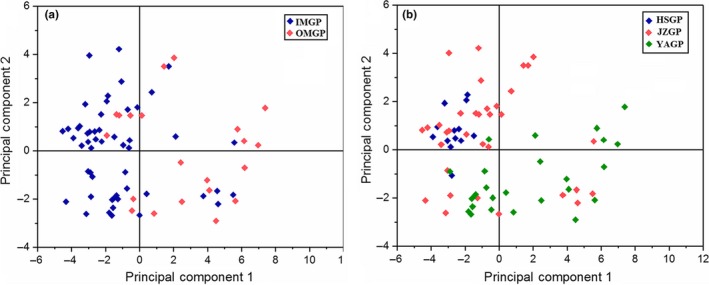
Principal component analysis (PCA) scoring plots for IMGP, OMGP (a) and JZGP, YAGP, HSGP (b)

Principal component analysis, as one of the commonly used multivariate analysis methods used for preliminary classification (Chen et al., 2015), was applied to extract the factors associated with production regions. Two principal components, explaining up to 96.87% of total variance, were revealed. Scoring plots are presented in Figure 4, with Figure 4a,b showing the classification of Group 1 and Group 2, respectively. Among them, PC1 explained 92.59% of the total variance, with variation mainly in GA, EGCG, and ECG contents; by contrast, PC2 explained 4.28% of the total variance, with variation mainly in EGC, C, and ECG contents. The distinction between the inner mountain and the outer mountain is more obvious, but the county area is mixed and the catechins seem to play a major role from the results of PCA. HCA and PCA are two methods of unsupervised pattern recognition, which obtained the preliminary classification information. The further visualized analysis of samples by combining supervised pattern recognition SVM is necessary.

### 3.4 Factor selection according to HCA, correlation analysis, and PCA

The final purpose of this study was to analyze the difference and discriminate LAGP from different origins using main chemical components combining pattern recognition. The results of factor selection are listed in Table 3. The selection upshot of HCA depends on the relative magnitude of the Euclidean distance, and PCA relies on the coefficient values of the variables that make up PC1 and PC2, especially.

**Table 3:** Results of factor selection and optimization

Methods	Screening criteria	Factors	
		Inner and outer mountains	Counties
Original factors	All components being tested	Polyphenols, amino acids, CAF	Polyphenols, amino acids, CAF
HCA	Euclidean distance	C, EC, ECG, Asp, Glu, Arg, Thea	EGC, C, Caf, EC, ECG, Thea, Arg, Asp, Glu
Correlation analysis	Pearson correlation coefficient	EGCG, Asp, Phe, Thr, Ala, Cys, Ser, Tyr, Val, Lys, Ile, Leu	GA, EGCG, ECG, Ser, Phe, Thr, Tyr, Val, Met, Ile, Leu
PCA	The principal components	GA, C, EGC, EGCG, ECG	GA, C, EGC, EGCG, ECG

### 3.5 Geographical tracing of LAGP through SVM combining with different factors

Support vector machine combining with the method of original variables and the factors selected by HCA, correlation analysis, and PCA all realized high discrimination statistically except for correlation analysis (Table 4). The results revealed that the model could obtain a high identification rate combining with PCA. The training and prediction sets of the correct classification for inner and outer mountains were 97.96% and 95.83%, with only two samples discriminated incorrectly. However, the discrimination between counties was worse than for IMGP and OMGP; the correct discrimination rates of the training set and prediction set were 91.84% and 95.83%. These results indicated that distinguishing LAGP by using SVM is feasible.

**Table 4:** Results of SVM with different factors

Samples	Factors selected (amount)	Correct discrimination rate	
		Training set	Prediction set
Inner and outer mountains	Origin (25)	100% (49/49)	91.67% (22/24)
	Origin—HCA (7)	100% (49/49)	95.83% (23/24)
	Origin—correlation analysis (12)	97.96% (48/49)	83.33% (20/24)
	Origin—PCA (5)	97.96% (48/49)	95.83% (23/24)
Counties	Origin (25)	93.88% (46/49)	95.83% (23/24)
	Origin—HCA (9)	87.76% (43/49)	87.50% (21/24)
	Origin—correlation analysis (11)	91.84% (45/49)	79.17% (19/24)
	Origin—PCA (5)	91.84% (45/49)	95.83% (23/24)

The geographical origins of tea are difficult to trace because of the many associated factors necessary to determine origin. The composition of contained matter of tea can be easily changed by climate (Wang et al., 2011), agricultural practices in tea plantations (Wang et al., 2016), and tea processing (Huang, Sheng, Yang, & Hu, 2007). Of these, geographical location is one of the most critical factors. The discrimination rate in this study between the inner and outer mountain samples was high, possibly because of the relationship with altitude exhibited by the samples (Table S1). In addition, slight discrepancy of parameter caused by working operations among different production areas might affect geographical tracing, too. Huoshan County and Jinzhai County are traditional LAGP processing areas that emphasize “heat” during processing, which makes JZGP and HSGP highly dissimilar to YAGP and might also explain the reason that identification of YAGP was easier.

## 4 CONCLUSIONS

Some studies have examined geographical tracing of tea products in terms of tea categories or tea‐planting areas. This study shows that the analytical information extracted from chemical components and the methodof pattern recognition can be used to discriminate the origin of LAGP. Hence, 25 component contents and their combinations with differing multivariate classifiers (HCA, correlation analysis, and PCA) were selected. Of these, Ile, Phe, Val, Leu, and Ser were key factors in the classification of IMGP and OMGP. Furthermore, GA and Met might be useful in classification of counties. The best result in classification was obtained by using SVM and PCA, which reached 95.83% classification accuracy in prediction set of inner and outer mountain and counties samples, respectively. The proposed method successfully achieved origin discrimination of LAGP without sensory evaluation. However, any generalization of the proposed methodology requires more extensive and varied testing of LAGP samples with tea samples from numerous years.

## CONFLICT OF INTEREST

Huan Su, Weiquan Wu, Xiaochun Wan, and Jing ming Ning declare that they do not have any conflict of interest.

## ETHICAL REVIEW

This study does not involve any human or animal testing.

## INFORMED CONSENT

Written informed consent was obtained from all study participants.
