# Supplementary material for: Discriminating geographical origins of green tea based on amino acid, polyphenol, and caffeine content through high‐performance liquid chromatography: Taking Lu’an guapian tea as an example
Source: Food Sci Nutr. 2019 May 16;7(6):2167–75. doi: 10.1002/fsn3.1062 (PMC6593377; doi:10.1002/fsn3.1062)
Supplement: Supplementary file 1 [file FSN3-7-2167-s001.docx]

**Table S1** Description of tea samples

| Origin level | Name | Shot name | County administration | Amount | Altitude(m) |
| --- | --- | --- | --- | --- | --- |
| Inner mountains | Eighteen curves | EC | Huoshan County | 7 | 350 |
|  | Da Pingdi | DPD | Huoshan County | 7 | 380 |
|  | Qishan Village | QM | Jinzhai County | 7 | 363 |
|  | Qiyun Village | QY | Jinzhai County | 6 | 231 |
|  | Flower Hill | FH | Jinzhai County | 7 | 210 |
|  | Du Hill | DH | Yu'an Distract | 7 | 188 |
|  | Shajia Cove | SJ | Yu'an Distract | 5 | 160 |
|  | Mashi Village | MS | Jinzhai County | 7 | 326 |
| Outer mountains | Shi Banchong | SBC | Yu'an Distract | 7 | 74 |
|  | 29 kilometers | 29K | Yu'an Distract | 7 | 90 |
|  | You Fangdian | YFD | Jinzhai County | 7 | 95 |

**Table S2** The content of each substance at 11 sampling points (WT%, means±SDs)

|  | EC | DPD | QM | QY | FH | DH | SJ | MS | SBC | 29K | YFD |
| --- | --- | --- | --- | --- | --- | --- | --- | --- | --- | --- | --- |
| GA | 0.052±0.006 | 0.045±0.009 | 0.022±0.006 | 0.051±0.010 | 0.041±0.009 | 0.019±0.005 | 0.029±0.008 | 0.042±0.007 | 0.029±0.011 | 0.029±0.011 | 0.027±0.006 |
| EGC | 3.569±0.101 | 2.835±0.195 | 2.529±0.028 | 3.326±0.247 | 3.173±0.271 | 3.574±0.181 | 3.746±0.432 | 2.725±0.225 | 3.710±0.240 | 2.401±0.129 | 2.191±0.088 |
| C | 0.047±0.001 | 0.079±0.003 | 0.127±0.008 | 0.146±0.003 | 0.144±0.009 | 0.042±0.001 | 0.067±0.000 | 0.132±0.010 | 0.048±0.003 | 0.136±0.002 | 0.064±0.003 |
| CAF | 3.339±0.285 | 3.568±0.154 | 3.538±0.117 | 4.747±0.143 | 4.077±0.188 | 2.912±0.184 | 3.070±0.150 | 3.948±0.198 | 3.163±0.193 | 3.604±0.169 | 2.754±0.250 |
| EC | 0.498±0.018 | 0.486±0.018 | 0.495±0.004 | 0.608±0.012 | 0.576±0.053 | 0.479±0.033 | 0.497±0.035 | 0.519±0.048 | 0.670±0.058 | 0.480±0.019 | 0.378±0.018 |
| EGCG | 11.968±0.68 | 10.019±0.511 | 8.822±0.494 | 11.658±0.715 | 9.559±0.522 | 7.869±0.667 | 8.820±0.431 | 8.167±0.226 | 8.124±0.500 | 7.508±0.551 | 5.802±0.389 |
| ECG | 1.794±0.123 | 1.818±0.105 | 1.944±0.122 | 2.549±0.095 | 2.112±0.197 | 1.231±0.035 | 1.364±0.068 | 1.971±0.117 | 1.473±0.107 | 1.571±0.116 | 1.206±0.102 |
| Asp | 0.106±0.010 | 0.112±0.004 | 0.104±0.011 | 0.118±0.011 | 0.155±0.009 | 0.145±0.001 | 0.136±0.011 | 0.141±0.011 | 0.189±0.012 | 0.147±0.013 | 0.215±0.012 |
| Ser | 0.034±0.002 | 0.038±0.003 | 0.038±0.000 | 0.039±0.000 | 0.053±0.003 | 0.045±0.003 | 0.05±0.001 | 0.055±0.001 | 0.096±0.002 | 0.088±0.001 | 0.107±0.005 |
| Glu | 0.146±0.014 | 0.152±0.011 | 0.136±0.013 | 0.147±0.015 | 0.171±0.011 | 0.189±0.013 | 0.153±0.011 | 0.187±0.010 | 0.157±0.011 | 0.156±0.013 | 0.204±0.015 |
| Gly | 0.003±0.000 | 0.003±0.000 | 0.003±0.000 | 0.003±0.000 | 0.004±0.000 | 0.003±0.000 | 0.003±0.000 | 0.003±0.000 | 0.003±0.000 | 0.004±0.000 | 0.003±0.000 |
| His | 0.030±0.001 | 0.034±0.001 | 0.029±0.001 | 0.031±0.002 | 0.037±0.002 | 0.036±0.002 | 0.037±0.010 | 0.046±0.001 | 0.039±0.003 | 0.043±0.001 | 0.052±0.002 |
| Arg | 0.084±0.006 | 0.106±0.010 | 0.058±0.022 | 0.085±0.037 | 0.094±0.028 | 0.081±0.018 | 0.090±0.020 | 0.187±0.034 | 0.067±0.025 | 0.114±0.042 | 0.182±0.077 |
| Thr | 0.015±0.002 | 0.014±0.001 | 0.013±0.002 | 0.014±0.001 | 0.018±0.001 | 0.016±0.001 | 0.016±0.001 | 0.020±0.001 | 0.033±0.002 | 0.032±0.002 | 0.027±0.001 |
| Ala | 0.017±0.002 | 0.016±0.002 | 0.017±0.001 | 0.017±0.001 | 0.022±0.001 | 0.020±0.003 | 0.018±0.002 | 0.022±0.001 | 0.025±0.001 | 0.024±0.001 | 0.025±0.001 |
| Pro | 0.053±0.008 | 0.049±0.007 | 0.054±0.002 | 0.046±0.005 | 0.054±0.001 | 0.050±0.001 | 0.041±0.004 | 0.053±0.001 | 0.057±0.001 | 0.061±0.001 | 0.062±0.007 |
| Cys | 0.008±0.000 | 0.010±0.001 | 0.008±0.001 | 0.007±0.001 | 0.008±0.001 | 0.009±0.001 | 0.009±0.001 | 0.010±0.001 | 0.010±0.003 | 0.013±0.001 | 0.011±0.001 |
| Tyr | 0.016±0.000 | 0.017±0.001 | 0.015±0.000 | 0.017±0.000 | 0.021±0.000 | 0.018±0.000 | 0.018±0.000 | 0.018±0.000 | 0.029±0.000 | 0.035±0.001 | 0.027±0.000 |
| Val | 0.008±0.000 | 0.009±0.001 | 0.009±0.000 | 0.011±0.000 | 0.014±0.000 | 0.011±0.000 | 0.012±0.000 | 0.011±0.000 | 0.029±0.000 | 0.029±0.000 | 0.028±0.000 |
| Met | 0.091±0.008 | 0.105±0.010 | 0.054±0.000 | 0.061±0.017 | 0.054±0.002 | 0.043±0.001 | 0.040±0.003 | 0.054±0.005 | 0.030±0.001 | 0.035±0.000 | 0.044±0.005 |
| Lys | 0.014±0.003 | 0.017±0.002 | 0.015±0.003 | 0.015±0.003 | 0.018±0.005 | 0.015±0.002 | 0.017±0.002 | 0.018±0.003 | 0.033±0.009 | 0.030±0.008 | 0.031±0.006 |
| Ile | 0.007±0.001 | 0.007±0.001 | 0.007±0.001 | 0.008±0.002 | 0.012±0.004 | 0.009±0.002 | 0.009±0.002 | 0.009±0.002 | 0.026±0.005 | 0.026±0.001 | 0.024±0.004 |
| Leu | 0.012±0.000 | 0.011±0.002 | 0.011±0.000 | 0.013±0.000 | 0.017±0.005 | 0.013±0.000 | 0.016±0.001 | 0.013±0.001 | 0.033±0.000 | 0.032±0.009 | 0.034±0.001 |
| Phe | 0.016±0.001 | 0.015±0.001 | 0.013±0.001 | 0.016±0.001 | 0.020±0.000 | 0.016±0.000 | 0.017±0.001 | 0.017±0.001 | 0.040±0.004 | 0.049±0.001 | 0.040±0.000 |
| Thea | 1.099±0.034 | 1.049±0.024 | 0.833±0.029 | 0.905±0.070 | 1.049±0.043 | 1.066±0.066 | 0.982±0.026 | 1.213±0.020 | 0.908±0.043 | 1.013±0.100 | 0.999±0.087 |
